# Bioavailability and Efficacy of a Gap Junction Enhancer (PQ7) in a Mouse Mammary Tumor Model

**DOI:** 10.1371/journal.pone.0067174

**Published:** 2013-06-12

**Authors:** Stephanie N. Shishido, Keshar Prasain, Amanda Beck, Thi D. T. Nguyen, Duy H. Hua, Thu Annelise Nguyen

**Affiliations:** 1 Department of Diagnostic Medicine and Pathobiology, Kansas State University, Manhattan, Kansas, United States of America; 2 Department of Chemistry, Kansas State University, Manhattan, Kansas, United States of America; University of Medicine and Dentistry of New Jersey, United States of America

## Abstract

The loss of gap junctional intercellular communication is characteristic of neoplastic cells, suggesting that the restoration with a gap junction enhancer may be a new therapeutic treatment option with less detrimental effects than traditional antineoplastic drugs. A gap junction enhancer, 6-methoxy-8-[(2-furanylmethyl) amino]-4-methyl-5-(3-trifluoromethylphenyloxy) quinoline (PQ7), on the normal tissue was evaluated in healthy C57BL/6J mice in a systemic drug distribution study. Immunoblot analysis of the vital organs indicates a reduction in Cx43 expression in PQ7-treated animals with no observable change in morphology. Next the transgenic strain FVB/N-Tg(MMTV-PyVT) 634Mul/J (also known as PyVT) was used as a spontaneous mammary tumor mouse model to determine the biological and histological effects of PQ7 on tumorigenesis and metastasis at three stages of development: Pre tumor, Early tumor, and Late tumor formation. PQ7 was assessed to have a low toxicity through intraperitoneal administration, with the majority of the compound being detected in the heart, liver, and lungs six hours post injection. The treatment of tumor bearing animals with PQ7 had a 98% reduction in tumor growth, while also decreasing the total tumor burden compared to control mice during the Pre stage of development. PQ7 treatment increased Cx43 expression in the neoplastic tissue during Pre-tumor formation; however, this effect was not observed in Late stage tumor formation. This study shows that the gap junction enhancer, PQ7, has low toxicity to normal tissue in healthy C57BL/6J mice, while having clinical efficacy in the treatment of spontaneous mammary tumors of PyVT mice. Additionally, gap junctional intercellular communication and neoplastic cellular growth are shown to be inversely related, while treatment with PQ7 inhibits tumor growth through targeting gap junction expression.

## Introduction

Gap junction intercellular communication (GJIC) has an important function in maintaining tissue homeostasis. GJIC is the process in which small metabolites are shared directly by contiguous cells that have their cytoplasms connected by aqueous channels called gap junctions. The loss of GJIC is related to the pathogenesis of multiple diseases, such as deafness and hearing loss, cataracts, skin disorders, oculodentodigital dysplasia, and cancer (see review [1]). The enhancement or restoration of functional gap junctions could be therapeutic treatment option for these diseases. Here we focus on the pathogenesis of cancer in relation to the loss of gap junction protein expression. A class of substituted quinolines was described in Shi et al. and the effects of the first generation compound (PQ1) as a gap junction enhancer in breast cancer cell lines has been explored [2] [3]. The second generation compound, 6-methoxy-8-[(2-furanylmethyl) amino]-4-methyl-5-(3-trifluoromethylphenyloxy) quinoline (PQ7), was shown to enhancer GJIC activity in cancer cells, with a more powerful effect on GJIC than the first generation PQ1 [4].

Many cancer treatment methods utilize chemotherapies that target mitotic cells for destruction, but this is not specific to the cancer cells and leads to severe side effects. The loss of GJIC by cancer cells is specific, suggesting that restoration of GJIC may provide a treatment with less detrimental effects to the host. Previous studies indicate that administration of PQ1 via oral gavage has a low toxicity to normal tissue of healthy C57BL/6J mice with no observable adverse effects [5], while significantly attenuating xenograft tumor growth of nude mice [6]. Here the distribution and anti-tumor effects of PQ7 are explored.

This study first determined the systemic distribution of PQ7 after intraperitoneal injection in healthy C57BL/6J mice. The drug distribution to the vital organs was determined at various periods of time after injection. Analysis using histological observation of PQ7 treated tissue showed no significant alterations in tissue organization or structure, suggesting a low toxicity. Next PQ7 was utilized as a treatment for mammary carcinoma in a spontaneous mammary tumor mouse model. The *in situ* generation of mammary tumors in the transgenic strain FVB/N-Tg(MMTV-PyVT) 634Mul/J (also known as PyVT) was used to determine the biological and histological effects of PQ7 on spontaneous tumorigenesis and metastasis. The PyVT model carries the Polyoma Virus middle T antigen with mammary tissue-specific expression driven by the mouse mammary tumor virus (MMTV) promoter [7]. Virgin females that carry the transgene develop poorly differentiated, multi-focal, invasive ductal carcinoma by 10–12 weeks of age, with a high incidence of lung metastases stemming from the primary mammary tumor [8]. Noninvasive focal lesions develop by 5 weeks and are classified into four groups: simple, solid, cystic, and mixed (solid and cystic) [9]. Development of tumors was divided into three time periods: Pre-tumor stage (up to 4 weeks old), Early tumor stage (6 to 8 weeks old) and Late tumor stage (more than 10 weeks old). For each stage, effect from PQ7 administration was evaluated and the expression of gap junction proteins was measured on treated tissue.

## Materials and Methods

### Ethics Statement

Husbandry of animals was conducted by the Comparative Medical Group (CMG) at the College of Veterinary Medicine at Kansas State University. The CMG animal facilities are fully accredited by the Association for Assessment and Accreditation of Laboratory Animal Care, International (AAALAC). The compliance to aspects of animal welfare law was regularly monitored by the veterinary staff. Animal care and use protocols were approved by the Institutional Animal Care and Use Committee (IACUC) at Kansas State University (Protocol Number: 2950), Manhattan following NIH guidelines.

### Compounds

PQ7,6-methoxy-8-[(2-furanylmethyl)amino]-4-methyl-5-(3-trifluoromethylphenyloxy) quinoline, was prepared as previously reported [2].

### Animals

Female C57BL/6J (The Jackson Laboratories, Bar Harbor, Maine 04609 USA) mice approximately 5 weeks of age were used in the distribution experiments. All mice were housed together in a temperature controlled environment (72 F) with a 12 hour light-dark cycle and unlimited access to standard mouse chow and water. Six animals were randomly assigned to each treatment group and administered 25 mg/kg PQ7 via intraperitoneal (IP) injection. At 6, 12, 24, and 36 hours post injection, all organs were harvested from animals euthanized by carbon dioxide inhalation.

A colony of FVB-TgN(MMTV-PyVT) transgenic mice (The Jackson Laboratory, Bar Harbor, ME) was established for mammary carcinoma studies. To identify transgenic progeny, genomic DNA was extracted from a 1.5-cm tail clipping. All mice carrying the PyVT transgene developed mammary tumors. Tumor development of positive female mice was closely monitored every 2–3 days. Tumor onset was recorded as the age of the animal at which palpable abnormal masses were detected. Tumor size was measured in two dimensions with calipers every 2 days as early as 5 weeks of age. Tumor volume was determined by the equation: Volume = ½(Length) *(Width)^2^. Mice were observed for any change in behavior, appearance, and weight. When animals reached a specific age range, six animals were randomly assigned to each treatment group and administered 25 mg/kg PQ7 via IP injection. Animal care and use protocols were approved by the Institutional Animal Care and Use Committee (IACUC) at Kansas State University, Manhattan, Kansas following NIH guidelines.

### PQ7 distribution studies in mice (HPLC and Mass Spectrometry)

#### Extraction of PQ7 from organs and plasma

Organs were cut into small pieces followed by the addition of 4 mL of deionized water and 10 mL of a solution of 9:1 ratio of ethyl acetate and 1-propanol. Plasma samples were directly mixed with 4 mL of water and 10 mL of a 9:1 solution of ethyl acetate and 1-propanol. Tissue and plasma solutions were separately sonicated for 40 minutes and 10 minutes, respectively, and the organic layer was separated from a separatory funnel. The aqueous layer was extracted twice with 10 mL of a 9:1 solution of ethyl acetate and 1-propanol. The organic layers were combined, washed with 5 mL of brine, dried over anhydrous MgSO_4_, and concentrated to dryness on a rotary evaporator. The residue was diluted with 1 mL of 1-propanol and filtered through a 0.2 µm filter disc (PTFE 0.2 µm, Fisherbrand) and analyzed using HPLC and mass spectrometry as described below.

#### Quantification of PQ7 using HPLC

HPLC analysis was carried out on a Varian Prostar 210 with a UV–vis detector and a reverse phase column (250 x 21.20 mm, 10 micron, Phenomenex, S. No: 552581-1). A flow rate of 5 mL/min and detection wavelength of 254 nm were used. A gradient elution of solvent A, containing deionized water and 0.01% of trifluoroacetic acid, and solvent B, containing acetonitrile and 0.01% of trifluoroacetic acid, was applied for the analysis.

1,2,4,5-Benzenetetracarboxylic acid (BTA) was used as an internal standard to quantify the amount of PQ7 in the tissue extracts. Solutions of 100 µL of various mixtures of authentic PQ7 and BTA were injected into a HPLC instrument, the peak areas corresponding to PQ7 and BTA were integrated from the HPLC chromatogram, and the ratios of the peaks were obtained. Results of the ratios of HPLC peak areas and ratios from PQ7 and BTA concentrations were plotted, and a linear correlation line was obtained from the graph. Hence using this correlation diagram, the ratio of HPLC peak areas of PQ7 and BTA from tissue extract, and the added known amount of BTA to the tissue extract, the amount of PQ7 in the tissue extract was determined.

Hence, 100 µL of 1:1 mixture by volume of the tissue or plasma extract and BTA of known concentration was injected into the HPLC, the peaks corresponding to PQ7 and BTA were integrated from the HPLC chromatogram, and the ratio of their masses was determined. Comparing the ratio of the masses of the peaks of PQ7 in the extract and standard BTA to the ratio of the masses of the peaks of authentic PQ7 and standard BTA, the mass of PQ7 in the organs and plasma was quantified.

#### Mass Spectroscopy

An Applied Biosystem API 2000 LS/MS/MS mass spectrometer was used in the analysis. The eluent corresponding to PQ7 peak from the HPLC was collected and injected into the mass spectrometer. A mass of 406 corresponding to M+1 of PQ7 was found in the mass spectrum, and the fragmentation pattern of this M+1 mass is similar to that of the authentic PQ7 verifying the identity of PQ7.

#### Antibodies

Primary antibodies: Anti-Cx46 (sc-20859, goat polyclonal), anti-PKCα (sc-8393, mouse monoclonal), and anti-Cx43 (sc-13558, mouse monoclonal), from Santa Cruz Biotechnology (Santa Cruz, CA); anti-GAPDH (2118, rabbit monoclonal) from Cell Signaling (Boston, MA) were used for both western blot and immunohistochemistry (IHC).

#### Western Blot Analysis

Mammary gland tumor tissue and selected organs (heart, lung, liver, spleen, kidney, uterus, brain) were homogenized in 500 mL of lysis buffer (20 mM Tris pH 7.5, 0.5 mM EDTA, 0.5 mM EGTA, 0.5% Triton X-100) with 1:1,000 dilution of protease inhibitors (Sigma-Aldrich, Saint Louis, MO). Tissue was homogenized via the OMNI Bead Ruptor 24 (Omni International, Kennesaw, GA) at a speed of 5.65 m/s for 45 seconds, followed by centrifugation at 13,000 rpm for 30 minutes at 4^°^C. Twenty-five µg of whole-cell extract was resolved by 10% SDS polyacrylamide gel electrophoresis (PAGE) and transferred to nitrocellulose membrane (Midwest Scientific, Saint Louis, MO). Nitrocellulose membrane was blocked in 5% milk for an hour at room temperature and then incubated with monoclonal antibodies (1:1,000). Western blots were detected by enhanced chemiluminescence detection reagents (Pierce, Rockford, IL) and visualized by Fluorochem E imaging system (ProteinSimple, Santa Clara, CA).

#### Immunohistochemistry (IHC)

Mammary carcinomas and organs were removed and fixed in a solution of 10% formaldehyde and embedded into paraffin prior to sectioning onto slides at a 5 µm thickness. Paraffin sections (5 µm) were dried at 60°C for 25 minutes. Deparaffinization was performed in solutions of 100% xylene and 100%, 90%, 75%, 50% ethanol. Antigen retrieval was performed in a steam chamber with 1× citrate buffer solution. Slides were then incubated overnight at room temperature with a polyclonal antibody (1:50 dilution). After washing in PBS, slides were successively incubated with biotinylated secondary antibodies (1:1,000) for 15 minutes. Slides were washed and immunostains were amplified by incubation with Avidin Biotin Complex (ABC) for 10 minutes. Cells were visualized with 3,3-diaminobenzidine (DAB) followed by a hematoxylin counterstain. The sections were viewed and the images captured with a Nikon 80i microscope under 40X and 60X magnification.

#### Statistical Analysis

Significance was determined at a P-value ≤ 0.05. Data is presented as the mean ± the 95% confidence interval of a minimum of three samples per treatment group.

## Results

### Distribution of PQ7

**Figure 1:**
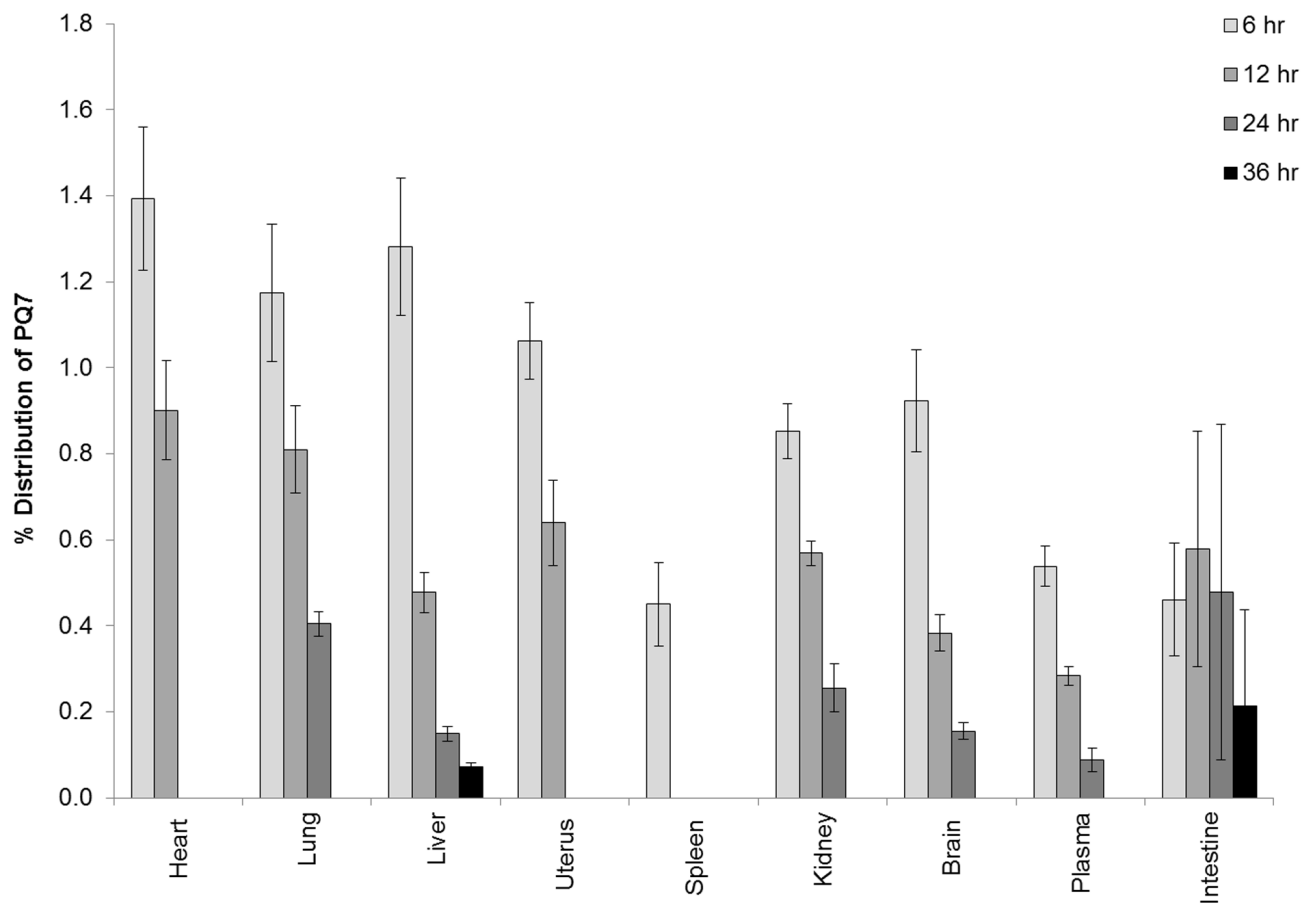
Distribution of PQ7 in mice. Mice treated with 25 mg/kg of PQ7 were euthanized at 6, 12, 24, and 36 hours. The total amount of PQ7 administered to each animal was defined as 100%. Bar graph represents the mean distribution of PQ7 with a 95% confidence interval. Data obtained from sample size of n = 6 mice.

Knowledge about the distribution of PQ7 in a biological system is important for the potential usage of this compound as an anticancer agent. PQ7 at 25 mg/kg was administered to 5-week-old female mice systemically by intraperitoneal injection. The total amount of PQ7 administered to each animal was defined as 100%. Six hours after the injection of PQ7, only 8.14% of the compound was detectable in the tissue collected. At 12, 24, and 36 hours post administration 4.65, 1.53, and 0.29% of the original compound was measurable by HPLC, respectively. Six hours after treatment the majority of PQ7 was detected in the heart, liver, lung, and uterus at levels of 1.4% (107 µM), 1.3% (98.74 µM), 1.2% (90.90 µM), and 1.1% (82.02 µM) of the total amount administered, respectively (Figure 1). A lower detectable level was measured in the kidney (0.85%; 65.94 µM) and brain (0.92%; 71.34 µM). At 12 hours post exposure, the concentration of PQ7 changed in the liver from 1.28% of that administered at 6 hours post injection to 0.47% (34.73 µM). At this time point PQ7 was no longer detectable in the spleen. At 24 hours post injection the compound was no longer detectable in the heart or uterus, while the lung and intestine had the highest concentration, at 0.41% (31.83 µM) and 0.48% (38.05 µM) respectively. After 24 hours of treatment, no PQ7 was found in the majority of the organs tested or the plasma. At 36 hours post exposure, the compound was detectable in limited amounts in the intestine (0.21%; 15.01 µM) and liver (0.07%; 5.21 µM). The trend in distribution of PQ7 remained fairly consistent in all tissues tested including plasma.

### Analysis of vital organs post PQ7 exposure

Multiple vital organs (brain, heart, liver and kidney) were examined using histopathology to determine any potentially detrimental effects of PQ7 administration in a single dose or in 7 doses spread over a period of 14 days. There were no morphological changes, evidence of hemorrhage, or inflammation in the tissues compared to control. This indicates that PQ7 had no toxicity to the normal tissue of healthy C57BL/6J mice. All mice exposed to PQ7 had no observed adverse effects on their health or behavior.

**Figure 2:**
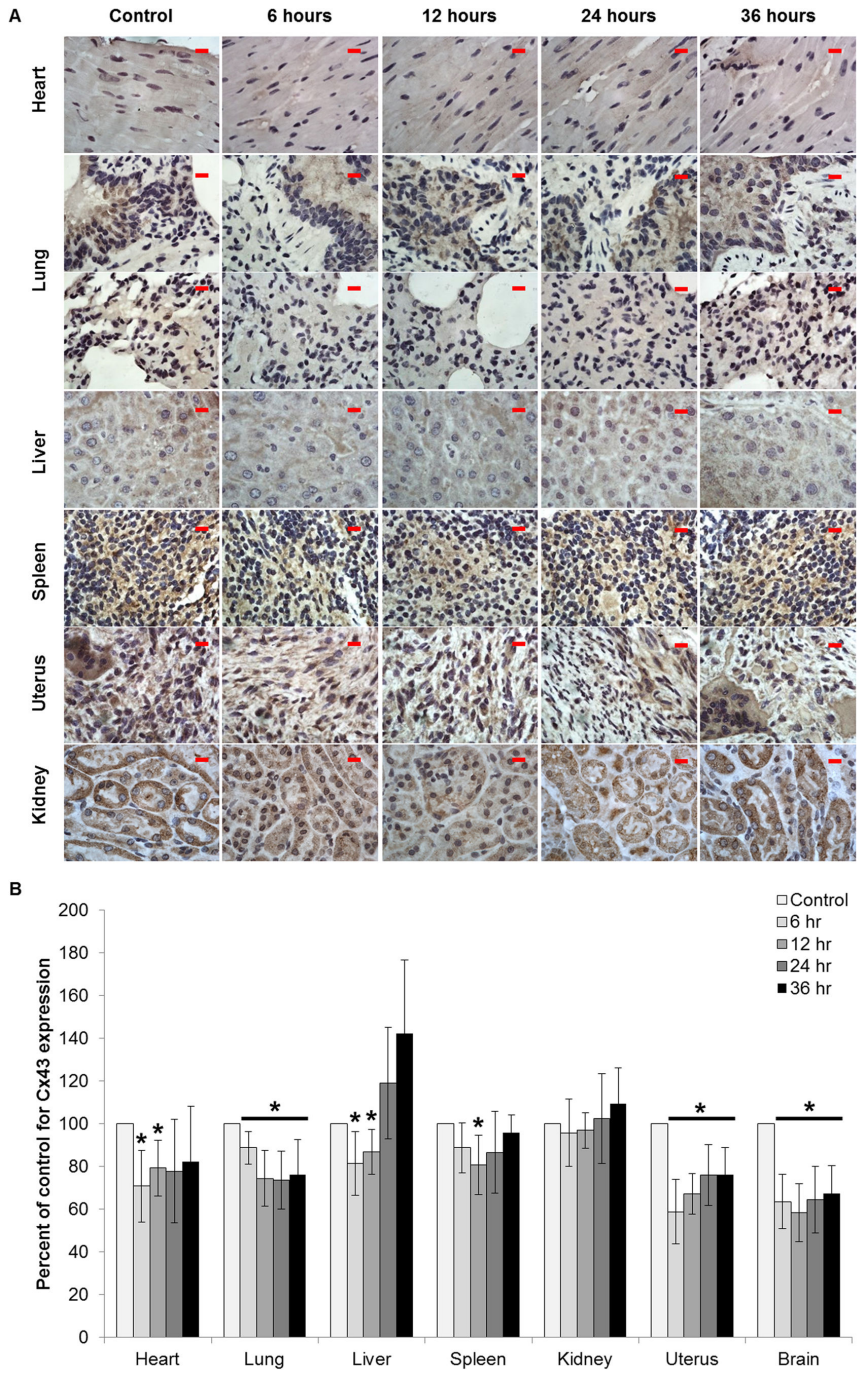
Effect of PQ7 on connexin 43 expression in normal tissue. A) Immunohistochemisty of tissue sections. Paraffin-embedded sections stained with antibodies against the gap junction protein Cx43in female C57BL/6J organs harvested after a single IP injection of PQ7 (25 mg/kg) at 6, 12, 24, and 36 hours. Proteins staining: brown, counterstaining: blue (hematoxylin). Images represent only 1 of n = 6 per group at a 100X magnification. Scale bar = 10 µm. B) Graphical representation of western blot analysis examining the effect of 6, 12, 24, and 36 hours of PQ7 treatment on the level of Cx43 expression. Mice without PQ treatment were used as a control. Bar graph shows the pixel intensities of protein bands normalized to the pixel intensities of loading control protein (actin) as a percentage of the control tissue. * P-value < 0.05 compared to control.

PQ7 has been shown to enhance GJIC and increase the expression of connexins (Cx) in neoplastic cells [4,6]. The expression of Cx43 in PQ7 treated and untreated organs were compared. Cx43 was detected in all tissues tested (Figure 2A). PQ7 treatment initially decreased Cx43 expression in the heart, lung, liver, uterus, and brain at 6 hours post injection (Figure 2B). The spleen had a significant decrease in Cx43 expression at 12 hours post injection. The heart and liver recovered normal expression levels after 24 hours. Cx43 expression in the lung, uterus, and brain remained significantly lower than normal over the 36 hours observed. There was no observable side effect due to the decreased expression levels. The kidney did not have a change in Cx43 expression.

### Effect of PQ7 on tumor growth in a spontaneous mammary tumor model

FVB-TgN(MMTV-PyVT) female transgenic mice developed tumors as early as 5 weeks of age and reached the maximum tumor burden around 15 weeks of age. Tumor development was divided into 3 stages based on the extent of tumor size, the frequency of tumor formation, and the presence of lung metastasis. The Pre stage of PyVT tumor development occurred at approximately 4-5 weeks of age, consisting of a pre-cancerous condition where no tumors were palpable and the mammary tissue appeared normal on gross observation. The Early stage of development represented solid tumor formation within the breast tissue with the gross observation of 1-2 solid tumors between 6–8 weeks of age. The Late stage occurred after 10 weeks of age and consisted of the presence of all 10 primary mammary tumors and secondary lung metastasis. The presence of metastases to the lung was confirmed by hematoxylin and eosin (H&E) staining of representative sections of the tissue followed by histopathological review.

**Figure 3:**
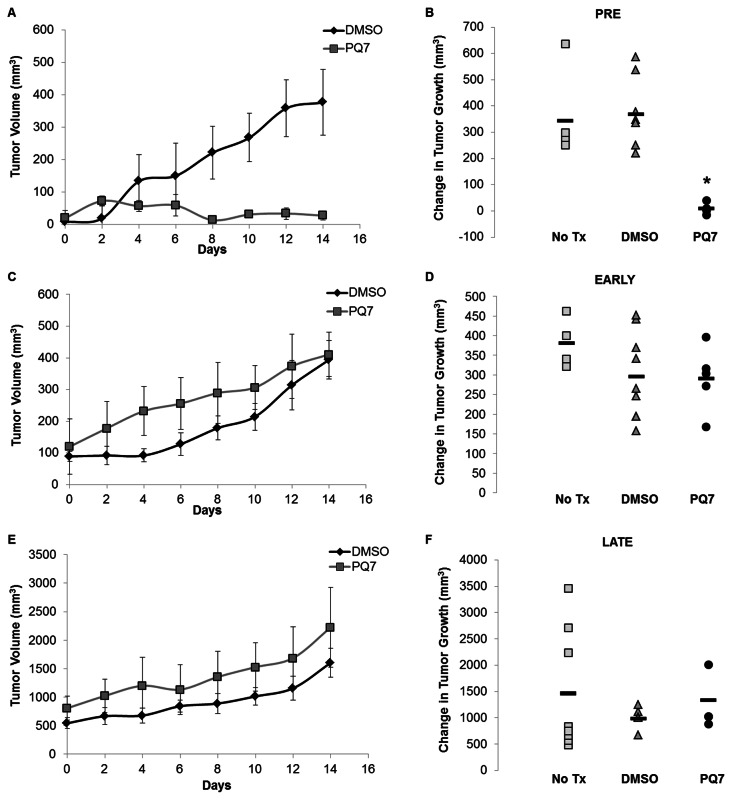
Tumor growth (mm^3^) in PyVT female mice. Tumors measured in two dimensions with calipers every 2 days prior to administration of treatment for panels A and B) Pre, C and D) Early, and E and F) Late stages of tumor development. Panels A, C, and E) The tumor size is expressed over the 14 day treatment period for the DMSO (control) and PQ7 (25 mg/kg) treated PyVT mice. Days 0-12 represent the days of the 7 IP injections, day 14 represents the end of the study with measurements prior to tissue harvest. Panels B, D, and F) The overall change in tumor size after no treatment, or treatment with DMSO (control) or PQ7 (25 mg/kg) via 7 IPs. * P-value < 0.05 compared to controls.

Tumor growth over a 14-day period with 7 IP injections of PQ7 or DMSO indicated a significant effect of PQ7 treatment on the Pre stage of neoplastic development in female PyVT mice. The initial tumor volume for all pre stage mice was 14.27 ± 13 mm^3^. There was a significant difference in tumor volumes between PQ7 and DMSO treated mice during the Pre stage of development from day 8 to day 14 (Figure 3A). PQ7 significantly attenuated tumor growth with a final volume of 27.8 mm^3^ over the 14-day treatment period (P-value = 0.0008). The final tumor growth of the control DMSO treated mice was 377 mm^3^. The change in tumor volume over the 14-day period shows a significant attenuation of tumor size with PQ7 treatment compared to both controls (P-value_NO TX_ = 0.005, P-value_DMSO_ = 0.0005; Figure 3B). There was a 98% difference between the overall changes in tumor growth after treatment with PQ7.

The initial tumor volume for all Early stage mice was 104 ± 53 mm^3^. During this stage of development there was not a significant difference in tumor growth between treatment groups (Figure 3C and 3D). During the Late stage of tumor development, mice began treatment with the initial tumor volume of 676 ± 134 mm^3^. PQ7 did not attenuate tumor growth compared to control during the Late stage of development (Figure 3E and 3F).

**Figure 4:**
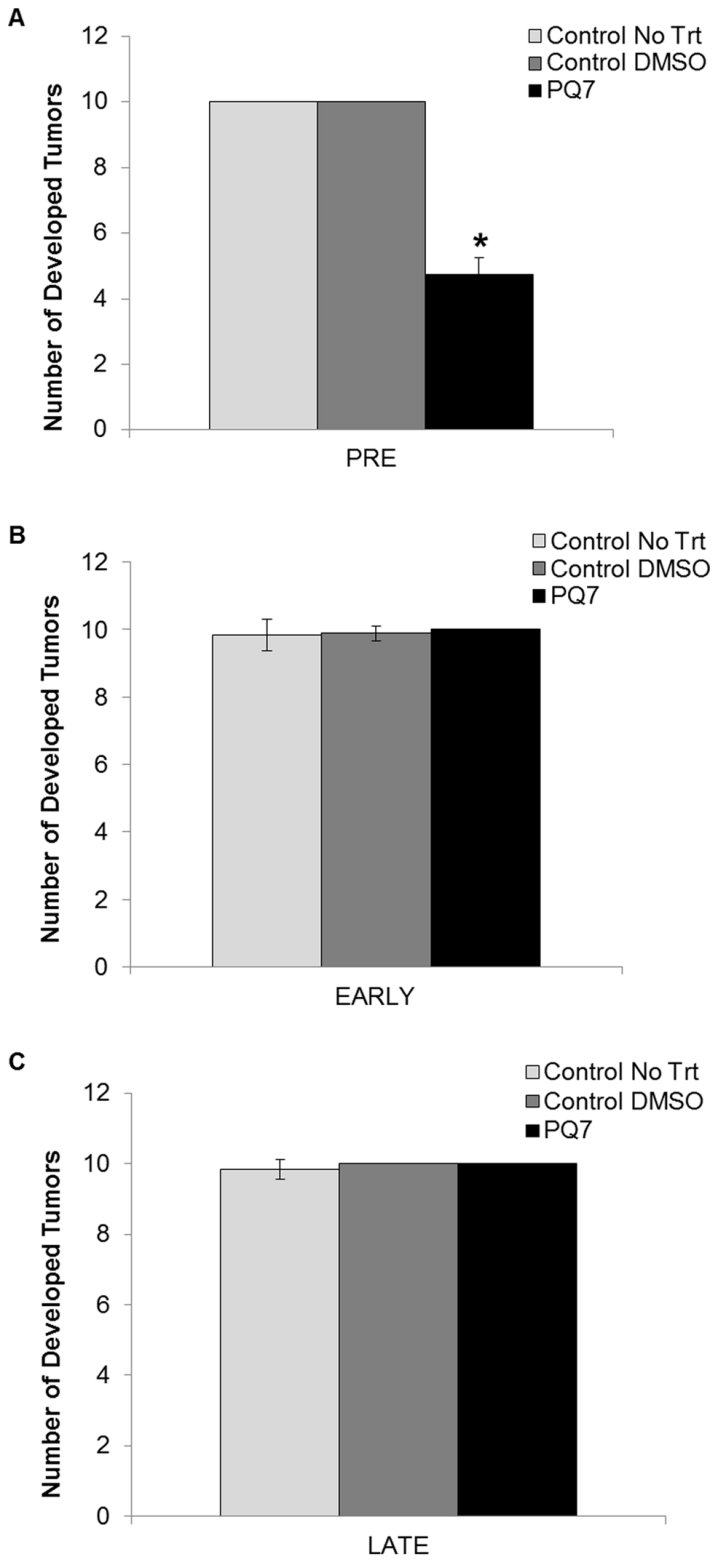
Number of developed tumors in PyVT female mice during development. Tumors identified grossly during the A) Pre, B) Early, and C) Late stages of tumor development after a 14 day period with either no treatment, or treatment with DMSO (control) or PQ7 (25 mg/kg) via 7 IPs. * P-value < 0.05 compared to controls.

PyVT mice have a total of 10 mammary fat pads that may develop tumors during their lifetime. The total number of palpable tumors, defined as the tumor burden, was monitored during the course of treatment, and the final tumor number for each treatment group in each stage of development is presented (Figure 4A–C). During all three stages there was no significant difference between the tumor burdens of the two control groups. Treatment with PQ7 during the Pre stage significantly reduced the number of tumors developed after treatment (P-value < 0.00001; Figure 4A). There was no difference in the tumor burden between experimental groups of the Early or Late stages of tumor development (Figure 4B and 4C).

**Figure 5:**
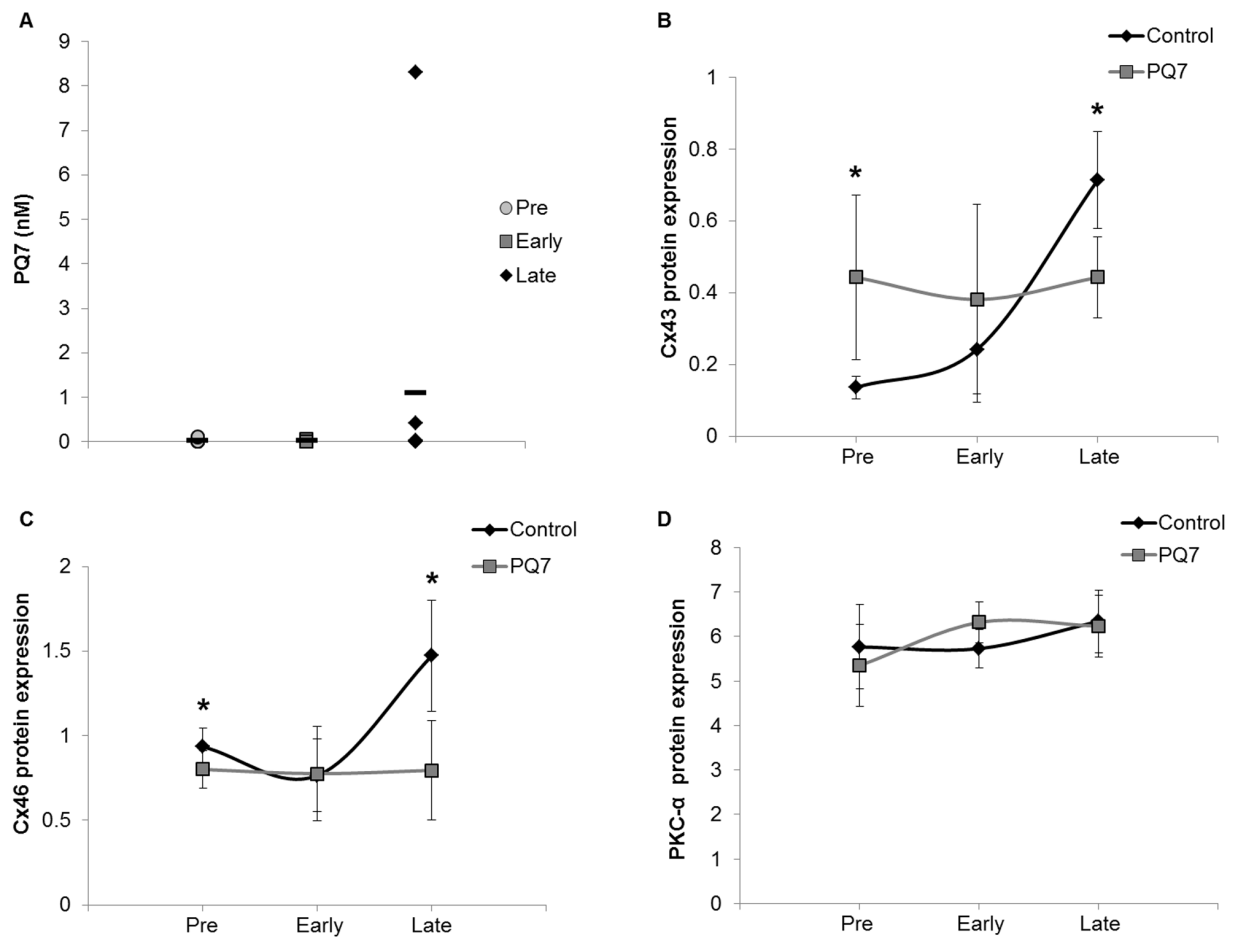
Analysis of tumors isolated from PyVT females 48 hours after the last IP injection. A) Quantitative analysis of PQ7 in the tumor homogenate. Data obtained from a minimum of three samples per developmental period. Data points represent the nanomolar concentration of PQ7 in each tumor isolated from treated mice, while the dashed lines represent the mean concentration of the PQ7 in all the tumors analyzed. B, C, and D) Graphical representation of protein expression in tumors from Western blot analysis. Fold-pixel intensity of B) Cx43, C) Cx46, and D) PKC-α normalized to loading control in PyVT female tumors treated with DMSO (control) or PQ7 (25 mg/kg) via 7 IPs in each of the three stages of tumor development. n = 4. * P-value < 0.05 compared to control.

Tumors were analyzed to determine the quantity of PQ7 detectable after approximately 48 hours after the last IP injection. At each stage of development, the parent compound was measurable in the neoplastic tissue harvested from treated animals. The Pre and Early stages of tumors were determined to have a concentration of 37 pM PQ7, while the Late stage tumors had 1.1 nM PQ7 (Figure 5A). This indicates that the parent compound remained in the tumor for at least 48 hours after a 14 day treatment period with 7 IP injections.

### Pathological analysis of PyVT tumors post PQ7 treatment

Histopathological examination of the mammary tumors of PyVT mice was conducted for each treatment group in the three stages of tumor development. When present, tumors were categorized as adenoma/mammary intraepithelial neoplasia (MIN), early carcinoma, or late carcinoma. Adenoma/MIN involved expansion of acini and ducts by a proliferation of polygonal neoplastic epithelial cells with multifocal coalescence of the affected ducts and acini. Neoplastic cells exhibited minimal cellular atypia and a low mitotic index (0-2/40x field). The neoplastic proliferation was confined by the basement membrane and there was a lack of fibrous connective tissue within the neoplasm. Early carcinomas were unencapsulated and moderately well-demarcated, with closely packed nests and acini of neoplastic cells with mild to moderate cellular atypia and 1-3 mitotic figures per high powered field. Neoplastic cells breached the basement membrane and were multifocally separated by a small to moderate amount of fibrovascular stroma. Late carcinomas were unencapsulated, poorly demarcated and invasive, composed of sheets of tightly packed nest and acini of neoplastic cells separated by moderate amounts of fibrovascular stroma. Anisocytosis and anisokaryosis were moderate and mitoses averaged 1-3/40x field. Adenosquamous carcinomas were late carcinomas with squamous differentiation.

The Pre control tumors were either adenoma/MIN or early carcinomas; while the Pre PQ7-treated tumors appeared to be focal hyperplasias or adenoma/MIN and early carcinoma. The Early control tumors were all early carcinomas. The Early PQ7-treated tumors varied from adenoma/MIN, early carcinoma, and late carcinoma. The Late control and PQ7 tumors were both late carcinomas. In addition a few Late PQ7 tumors were identified as adenosquamous carcinomas. Histological examination of the lung tissue from all Late stage mice showed no significant difference in the presence of metastatic foci between treatment groups.

### Effect of PQ7 on connexin expression in neoplastic tissue

PQ7 has been shown to enhance GJIC and increase Cx43 expression in breast cancer cells [4], therefore the differential pattern of connexin proteins in PQ7-treated tumors was determined. Though most connexins are tumor suppressors, Cx46 has been shown to be upregulated in breast cancer cell lines and tumors to provide protection in hypoxic conditions [10]. Immunoblot analysis of connexin expression indicates that PQ7 treatment increased Cx43 expression (Figure 5B) and decreased Cx46 expression (Figure 5C) during the early stages of carcinogenesis. During the control PyVT tumor development there was an increase in Cx43 and Cx46 expression from Pre to Late stage. Data suggests that the gap junction protein connexin 43 was expressed at higher levels in PQ7-treated animals compared to controls and the contrary for connexin 46 early in tumor formation. Cx46 expression in PyVT tumors treated with PQ7 from the Pre and Late stages of development had significantly lower levels than that of the controls (P-value_Pre_ = 0.016, P-value_Late_ = 0.0007). The Pre stage tumors treated with PQ7 had a significantly greater level of Cx43 expression compared to controls (P-value_Pre_ = 0.040), while during the Late stage they had significantly less Cx43 compared to controls (P-value_LATE_ = 0.034). This may be explained by the overall increase in both connexin 43 and connexin 46 during tumor development and metastasis of the PyVT mice.

**Figure 6:**
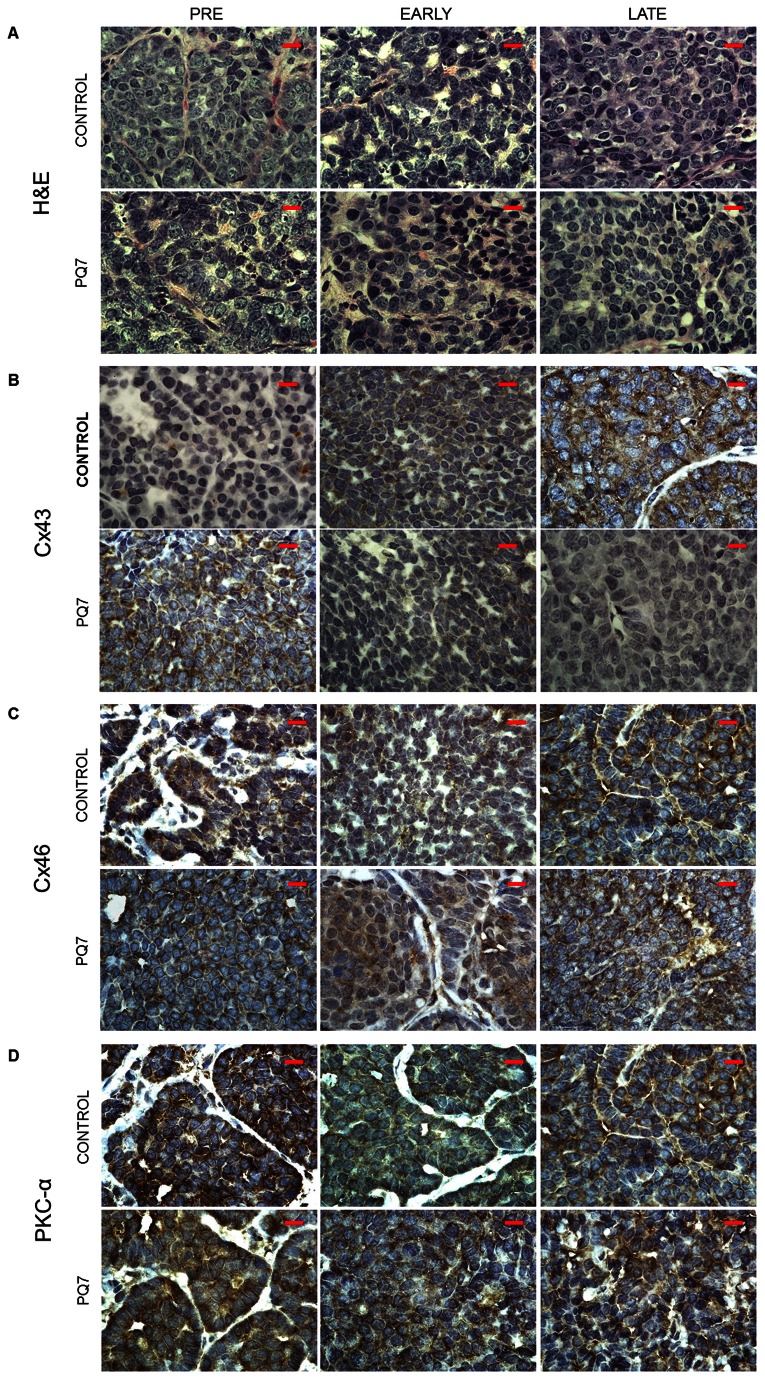
Immunohistochemisty of tumors from PyVT females. Paraffin-embedded tumor sections stained with A) H&E or antibodies against B) Cx43, C) Cx46, and D) PKCα from PyVT females treated with DMSO (control) or PQ7 (25 mg/kg) via 7 IPs at either Pre, Early, or Late stage of tumor development. Proteins staining: brown, counterstaining: blue (hematoxylin). Images represent only 1 of n = 6 per group at a 100X magnification. Scale bar = 10 µm.

Histopathology of the harvested PyVT tumors showed no significant difference in morphology. Immunohistochemistry of PQ7 treatment at Pre and Early stages of tumor formation showed stronger positive cytoplasmic staining in Cx43, while during the Late stage there was stronger positive staining in the control tissue versus the PQ7 treated tissue (Figure 6A). The Cx46 immunohistochemistry indicated a weaker positive staining compared to controls. This supports the molecular analysis previously mentioned.

Gap junction proteins are phosphoproteins that are targeted by kinases for efficient trafficking, assembly and disassembly, degradation, and gating of hemichannels [11–13]. Phosphorylation regulates GJIC in both a kinase and connexin specific manner [11,14]. Since PQ7 altered connexin expression, we explored the role of PKCα in the PQ7 treated PyVT tumors at each stage of development by western blot analysis (Figure 5D) and immunohistochemistry (Figure 6C). No significant change in PKCα expression was determined due to PQ7 treatment. Interestingly in the control tumors there appeared to be a stronger positive staining in the Pre stage compared to the Late stage of tumor development, suggesting a decrease in connexin phosphorylation and degradation.

## Discussion

Effective use of antineoplastic drugs depends on the ability to balance the killing of tumor cells against the inherent toxicity to the host. Antineoplastic agents that act primarily on rapidly dividing and growing cells produce multiple side effects and are dose limiting. The first generation gap junction enhancer was shown previously to have a low toxicity in healthy animals administered via oral gavage [5]. An intraperitoneal injection was used in this study to ensure a systemic exposure of a consistent amount of PQ7 to all the mice. Exposure of C57B/6J and PyVT mice to either a single or multiple doses, respectively, of PQ7 systemically showed no detrimental effects to any of the vital organs. Uptake of the compound into a specific tissue depends on the availability of the compound in the blood supply and the extent of vascularization. The highest levels of PQ7 were detectable in the liver, heart, lung, and uterus, which may be due to the fact that these are highly vascularized tissues. Interestingly PQ7 is detectable is the brain, indicating good penetration of the blood brain barrier. The 25 mg/kg PQ7 administered to approximately 20 g mice was equivalent to 9.56 mM. Results indicate that a 9.5 mM concentration of PQ7 can be distributed to all the vital organs and metabolized in C57B/6J mice.

Exposure of C57B/6J and PyVT mice to either a single or multiple doses, respectively, of PQ7 systemically showed no detrimental effects to any of the vital organs, indicating a low toxicity [5,7]. The total amount of PQ7 detected in the tissue after six hours was only 8.1%. This suggests that the majority of the parent compound is metabolized and/or eliminated in less than six hours. The half-life of PQ7 in the liver appeared to be about 6 hours, suggesting complete metabolism or elimination by 48 hours. The optimal time between injections for treatment purposes would therefore be 48 hours, which has been shown to be effects in tumor bearing mice [6].

The levels of PQ7 measured in the intestinal tract had a high variability, however the compound was detectable at the highest level in this organ 36 hours post exposure. The intestinal mucosal layer accumulates lipids and hydrophobic compounds, which have an increased permeability in the intestinal tract. This suggests that PQ7 may be secreted into the gastrointestinal tract through the bile duct for fecal excretion and potentially reabsorbed into the intestinal mucosa due to its lipophilicity. This is supported by the lack of PQ7 detected in the plasma or kidney after 24 hours, indicating that urinary excretion of the parent compound is complete by 24 hours post injection. Collectively these results suggest that PQ7 treatment may be useful in targeting neoplasias of the gastrointestinal tract.

The PyVT mouse is a novel *in vivo* model for mammary carcinoma formation and metastasis with important clinical utility. PyVT premalignant tumors are morphologically heterogeneous with highly proliferative neoplastic cells containing abnormal microvasculature and atypical nuclei, while remaining within the basement membrane [9]. The MMTV-PyVT expression is variable in tumors [9], which indicates that the transgene is not necessary for the maintenance of the malignancy, but only the initiation of the neoplastic cells. The PyVT model can be utilized as a multistage model of carcinogenesis due to advancing lesions from a pre-cancerous state of hyperplasia to an adenoma/mammary intraepithelial neoplasia mixed phenotype, followed by an early and late carcinoma with eventual pulmonary metastasis [8,9]. The formation of secondary tumors in the lung is advantageous for studying metastasis, which is a cause of death in many cancer types. Pathologically the neoplastic lesions are clinically similar to humans [9], stressing the value of this spontaneous model in this study.

Cell proliferation and apoptosis are important factors in carcinogenesis [15], and GJIC is a key factor in carcinogenic process. Reduced GJIC in preneoplastic and neoplastic tissue can lead to excessive cell proliferation, abnormal differentiation, and inhibited apoptosis, leading to the loss of homeostasis. More than 100 non-mutagenic and mutagenic carcinogens were reported to inhibit GJIC *in vitro* and *in vivo* [16–18]. These compounds are chemically diverse, including pharmaceuticals, polyaromatic hydrocarbons, plant products, and pesticides. The inhibition of GJIC correlates best with carcinogenicity in multiple *in vitro* tests [19]. This shows that the carcinogenic mechanism of multiple agents involves the down-regulation of GJIC. Therefore a compound that restores GJIC is vital for cancer prevention and treatment. The ability to normalize GJIC in neoplastic cell could restore homeostasis and prevent further tumor development.

Many tumor promoters down-regulate GJIC to allow the initiated cell to proliferate and evade apoptosis [20]. The down-regulation of GJIC is a reversible process, indicating that intervention that enhanced GJIC could prevent promotion and progression of the neoplastic tissue. Previously PQ7 was shown to increase the expression of gap junction proteins and enhance GJIC [3,4]. The data presented here shows that PQ7 delays the development of mammary carcinomas, suggesting it could be utilized as a primary chemopreventive compound for breast cancer. The PyVT mouse has a genetic alteration that predisposes them to the development of mammary carcinomas, however with PQ7 treatment during a pre-cancerous stage, the development of these malignancies was delayed significantly. The use of chemical intervention before an initiated cell becomes independent of the promoter stimuli could induce regression of the neoplastic tissue which is a process of chemoprevention.

Cancer is a prominent disease throughout the world, despite the increasing knowledge of carcinogenesis and treatment options. More effective cancer therapies are needed. Due to the fact that GJIC is involved in the development of cancer and metastasis, it is a promising target for new therapies. The enhancement of GJIC has been shown to increase the efficacy of multiple types of cancer therapies through the bystander effect [21–27]. GJIC could increase the distribution of chemotherapeutic compounds in tissues that are poorly vascularized and have impaired drug delivery, this is especially important for hydrophilic compounds that are unable to pass through the cell membrane [28]. Additionally, up-regulation of GJIC has been shown to increase the sensitivity of cancer cells to conventional chemotherapeutics [29,30]. Though PQ7 is not an effective anticancer compound on its own during later stages of tumor development, it could be used in combination with multiple types of chemotherapeutic options to enhance killing of the neoplastic cells.

Molecular analysis of the protein expression demonstrated a general increase of expression of Cx43 and Cx46 during tumor development. Cx46 is a hypoxia-specific gap junction protein in mammary tissue suggested to have pro-tumor effects by preventing hypoxic death [31]. As a tumor grows in size, the neoplastic cells in the center of the mass may upregulate Cx46 to survive more hypoxic conditions. Additionally an increase in Cx43 expression typically correlates with metastatic potential [32,33]. Interestingly expression of Cx43 in PQ7 treated animals has a reciprocal relationship with control Cx43 expression, while Cx46 expression in treated tissue remains low despite the stage of development. PQ7 affects the expression of each connexin differently during tumor development. Importantly the decrease in Cx46 and increase in Cx43 observed during the Pre stage of development with PQ7 treatment may be the key for prevention or delay of tumor formation. Additional knowledge of the role of each gap junction protein in tumorigenesis is needed.

The gap junction enhancer PQ7 is shown here to have no apparent side effects when systemically distributed to all the vital organs, and is capable of altering the development of a spontaneous mammary carcinoma. These results are promising in the development of a novel compound for chemoprevention or combinatory uses for breast cancer.
